# KpFhaB/FhaC is a virulence-associated TPS system of the globally-disseminated *Klebsiella pneumoniae* ST15 high-risk clone

**DOI:** 10.3389/fcimb.2026.1787622

**Published:** 2026-06-03

**Authors:** Ana Tajuelo, Eva Gato, Carlota Moya, Sonia Prieto Martín-Gil, Beatriz Cano-Castaño, Leilani Vaughan, Pedro Miguela-Villoldo, María Pérez-Vázquez, Miriam Moscoso, Bruno Kotska Rodiño-Janeiro, Félix Docando, María C. Terrón, Antonio J. Martín-Galiano, Michael J. McConnell, Germán Bou, Astrid Pérez

**Affiliations:** 1 Laboratorio de Infecciones Intrahospitalarias, Centro Nacional de Microbiología, Instituto de Salud Carlos III (ISCIII), Madrid, Spain; 2 Escuela internacional de Doctorado, Ciencias de la Salud, Universidad Nacional de Educación a Distancia (UNED), Madrid, Spain; 3 Servicio de Microbiología, Hospital Universitario A Coruña, Instituto de Investigación Biomédica de A Coruña (INIBIC), Universidad de A Coruña, A Coruña, Spain; 4 Laboratorio de Referencia e Investigación en Resistencia a Antibióticos e Infecciones Relacionadas con la Asistencia Sanitaria, Centro Nacional de Microbiología, Instituto de Salud Carlos III, Madrid, Spain; 5 Centro de Investigación Biomédica en Red de Enfermedades Infecciosas (CIBERINFEC), Instituto de Salud Carlos III, Madrid, Spain; 6 Electron Microscopy Unit, Central Core Facilities, Instituto de Salud Carlos III, Majadahonda, Madrid, Spain; 7 Unidad de Proteómica, Unidades Centrales Científico-Técnicas, Instituto de Salud Carlos III (ISCIII), Madrid, Spain; 8 Department of Biological Sciences, University of Notre Dame, Notre Dame, IN, United States; 9 Eck Institute for Global Health, University of Notre Dame, Notre Dame, IN, United States; 10 Departamento de Fisioterapia, Medicina y Ciencias Biomédicas, Universidad de A Coruña, A Coruña, Spain

**Keywords:** adherence, biofilm formation, FhaB/FhaC, *Klebsiella pneumoniae*, phylogeny, ST15

## Abstract

**Introduction:**

The global dissemination of *Klebsiella pneumoniae* sequence type 15 (ST15), a Q9 high-risk clone frequently resistant to carbapenems and third-generation cephalosporins, represents a major public health concern. While chaperone-usher pili (CUP) systems (i.e. Kpi system) are known mediators of K. *pneumoniae* adhesion and biofilm formation, additional adhesin mechanisms remain poorly characterized in this pathogen. In this study, we identified and functionally characterized a two-partner secretion (TPS) system, KpFhaB/FhaC, in the outbreak strain *K. pneumoniae* Kp3380 (ST15).

**Methods:**

Genomic and bioinformatic analyses were performed to identify and characterize the KpFhaB/FhaC TPS system. Knockout strains lacking fhaC, Kpi system, or both were constructed for functional studies. Biofilm formation, epithelial cell adhesion, bacterial fitness, and *in vivo* intestinal colonization were evaluated using *in vitro* and murine models.

**Results:**

KpFhaB/FhaC was identified as a conserved TPS system associated with the *K. pneumoniae* ST15 high-risk clone. It promotes biofilm formation and adhesion to human colorectal epithelial cells. Combined inactivation with the Kpi pili system produced an additive reduction in these phenotypes, indicating complementary roles in adhesion. Inactivation of KpFhaB/FhaC resulted in a significant fitness cost, whereas it did not significantly affect intestinal colonization *in vivo*.

**Discussion:**

These results suggest a functional specialization between both systems in *K. pneumoniae* ST15, suggesting that retaining them could give a selective advantage for this high-risk clone. Overall, our findings indicate that KpFhaB/FhaC is a conserved, chromosomally encoded TPS system that positively contributes to adhesion, biofilm formation and bacterial fitness in *K. pneumoniae* ST15.

## Introduction

The global spread of extremely drug-resistant *Klebsiella pneumoniae* represents a serious public health concern due to the expansion of a few high-risk clones (Wyres et al., 2020). Among these, sequence type (ST) 15 has emerged as a globally disseminated linage linked to healthcare-associated infections and rapid acquisition of multidrug resistance (Ewers et al., 2014; Markovska et al., 2015; Lam et al., 2019). The ST15 is now one of the most prevalent third-generation cephalosporin-resistant linages and is frequently linked to carbapenem resistance, a combination that prompted the World Health Organization (WHO) to designate carbapenem resistant *K. pneumoniae* as a critical-priority pathogen (WHO, 2024).

The ST15 strains typically harbor a wide array of resistance determinants, including extended spectrum betalactamases (ESBLs) such as *bla*_CTX-M-15_ and carbapenemases such as *bla*_KPC_, *bla*_NDM_ and *bla*_OXA-48-like_, often carried on plasmids that facilitate horizontal gene transfer both within *K. pneumoniae* and across bacterial species (Navon-Venezia et al., 2017; David et al., 2019). Beyond antimicrobial resistance, the success of ST15 is due to its superior ability to colonize the human gastrointestinal tract and persist in hospital settings, creating a reservoir for transmission (Gato et al., 2020).

The molecular mechanisms underlying the epidemic success of ST15 remain poorly understood. Given the limitations of therapeutic options against ST15, interrupting early events of infection, particularly bacterial adhesion and intestinal colonization, represents a promising strategy for controlling its spread (Wyres et al., 2020). Adhesion in *K. pneumoniae* is mediated primarily by chaperone-usher pili (CUP) systems, with type 1 (*fim*) and type 3 (*mrk*) pili present in nearly all isolates, and type 3 pili strongly promoting biofilm formation (Paczosa and Mecsas, 2016). *K. pneumoniae* genomes may contain loci encoding up to 10 distinct CUPs (Wu et al., 2010; Khater et al., 2015). Other adhesins, such as the plasmid-encoded factor KPF-28 and CF29K, contribute to intestinal adhesion (Favre-Bonte et al., 1995; Di Martino et al., 1996), and the type VI secretion system enhances colonization by modulating the expression of type 1 fimbriae (Hsieh et al., 2019).

Recent work has identified Kpi, a novel CUP system associated with ST15 and essential for gut colonization and biofilm formation (Gato et al., 2020). However, other adhesin pathways remain unexplored. Two-partner secretion (TPS) systems mediate epithelial adhesion in *Bordetella pertussis* through filamentous hemagglutinin (FHA) (Melvin et al., 2015; Scheller et al., 2015), enhance biofilm formation and virulence in *Acinetobacter baumannii* (Pérez et al., 2017), and promote intestinal colonization in enterotoxigenic *Escherichia coli* via the EtpA exoprotein (Roy et al., 2008). TPS systems typically involve a large exoprotein (TpsA) and outer-membrane translocator (TpsB) bearing polypeptide transport-associated (POTRA) domains (Van Ulsen et al., 2014).

Here we report the first functional characterization of the KpFhaB/FhaC TPS system in *K. pneumoniae*, using the outbreak strain Kp3380 as a model. This highly adherent strain, remarkable for its gastrointestinal colonization capacity and environmental persistence, provided us a platform to investigate the contribution of KpFhaB/FhaC to the pathobiology of the high-risk clone *K. pneumoniae* ST15.

## Materials and methods

### Bacterial strains, plasmids and growth conditions

The strains and plasmids used in this study are listed in **Table 1**. The clinical isolate *K. pneumoniae* Kp3380 (ST15, OXA-48 producer) was recovered at Hospital A Coruña, Spain. *E. coli* TG1 served as the cloning host. Strains were stored at –80 °C in LB broth with 15% (v/v) glycerol and cultured on MacConkey agar (BD) or in LB broth/agar at 37 °C. When required, media were supplemented with apramycin (200 µg mL^−1^), hygromycin (250 µg mL^−1^), or gentamicin (50 µg mL^−1^).

**Table 1:** Bacterial strains and plasmids used in the present study.

Strains and plasmids	Relevant characteristics	Reference or source
Kp3380	Outbreak-related *K. pneumoniae* ST15 isolated from wound exudate in 2015. Barcode AN2344 at bioproject PRJEB39112.	This study
Kp3380 derivative strains		
Kp3380Δ*kpiD*	*kpiD*-defective Kp3380 mutant strain	(Gato et al., 2020)
Kp3380Δ*fhaC*	*fhaC*-defective Kp3380 mutant strain	This study
Kp3380 Δ*kpiD*Δ*fhaC*	*kpiD* and *fhaC*-defective Kp3380 mutant strain	This study
Kp3380 Δ*fhaC*_C	Kp3380Δ*fhaC* strain harboring pUCP24/T_*fhaC*	This study
*E. coli* strains		
TG1	Host strain used for cloning procedures	Invitrogen
Plasmids		
pIJ773	Template vector for apramycin gene amplification	(Huang et al., 2014)
pACBSR-hyg	Plasmid (hygromycin) containing an arabinose-inducible 𝜆-Red recombinase	(Huang et al., 2014)
pFLP-hyg	Plasmid (hygromycin) containing a heat shock inducible FLP recombinase and a p15A replicon	(Huang et al., 2014)
pUCP24/T	Modified pUCP24 expression vector (tetracycline promoter)	(Gato et al., 2020)
pUCP24/T_*fhaC*	pUCP24/T containing the *kpiD* gene	This study

### Comparative genomics and phylogenetic analyses

A TPS locus, KpFhaB/FhaC, previously associated with ST15 in an outbreak of carbapenem-resistant *K. pneumoniae* (Gato, 2021; Gato et al., 2023), was interrogated by phylogenetic and population-level analyses. For cross-species comparisons, 41,728 *fhaBC*-related features were retrieved from the Bacterial and Viral Bioinformatics Resource Center (BV-BRC) database (https://www.bv-brc.org/; accessed 10 January 2025). All these features were annotated as exoproteins so the *fhaB* gene was selected as the reference for comparative analyses. Only the longest representative sequence (> 5 kb) per species was retained (n = 349) and aligned to *fhaB* of Kp3380 using MUSCLE v3.8.1551(maxiters = 2) (Edgar, 2004).

To assess the distribution of KpFhaB/FhaC system among European isolates, a custom ARIBA database was built from Kp3380 *fhaB* and *fhaC* sequences and screened against raw reads from 2,621 genomes included in EuSCAPE (ENA PRJEB10018/ERP011196), an European Survey on Carbapenamase-Producing Enterobacteriaceae. Raw reads were mapped to both genes to reconstruct the full-length *fhaB*, which often appears incomplete in assemblies result from extensive 20-residue filamentous hemagglutinin repeats. Assemblies < 5 kb were excluded, yielding 335 sequences for MUSCLE alignment. Maximum-likelihood phylogenies were inferred with IQ-TREE v2 (Trifinopoulos et al., 2016) using ModelFinder Plus (–m MFP) and branch support from 1,000 ultrafast bootstrap replicates plus SH-aLRT. Phylogenetic trees were visualized in iTOL v7 (https://itol.embl.de).

Presence/absence of *fhaC* was further assessed in 1,649 EuSCAPE *K. pneumoniae* genomes from 32 European countries by BLASTn using MegaBLAST, retaining hits with ≥ 97% identity and 100% coverage. The *fhaC* gene was used as a proxy due to the frequent incompleteness of *fhaB* in assembled genomes, which results from its repetitive structure. Core-genome MLST (cgMLST) was performed with 2,538 loci (Ridom SeqSphere+ v3.5) to compare *fhaC*-positive and *fhaC*-negative isolates.

### In silico analyses

Protein domains and motifs were identified with the InterPro server (PMID: 39565202). Protein homology was analyzed with the HHpred server (PMID: 29258817). Codon usage bias was evaluated with CodonW (http://codonw.sourceforge.net) using default parameters. Putative prophage regions and insertion sequences (IS) were searched using PHASTER (Arndt et al., 2016)and ISFinder (Siguier et al., 2006), respectively. Predicted Rho-independent transcriptional terminators were detected using iTerm- (Feng et al., 2019). The full cluster nucleotide sequence was queried by BLASTn against the NCBI core nucleotide (nt) database, masking the taxon *K. pneumoniae*, to identify homologous loci in genomes from other bacterial species.

### Construction of Δ*fhaC* mutants

A 771 bp internal fragment of *fhaC* was deleted from Kp3380 and its Kp3380Δ*kpiD* derivative following the protocol of Huang et al., with some modifications (Huang et al., 2014; Gato, 2021). An apramycin resistance cassette flanked by FRT sites was amplified from pIJ773 with primers carrying 60 bp homology to *fhaC* (**Supplementary Table 1**). Upstream and downstream flanking regions were amplified and fused by overlap PCR. The three fragments were assembled by two-step PCR, and the product was electroporated into Kp3380 or Kp3380Δ*kpiD* carrying pACBSR-Hyg. Apramycin-resistant colonies were screened by PCR and sequencing to confirm cassette insertion. Mutants were serially passaged on LB medium with or without apramycin (200 µg mL^−1^) and hygromycin (250 µg mL^−1^) to cure the helper plasmid.

### Complementation of Δ*fhaC*

The *fhaC* gene was amplified from Kp3380, digested with SmaI and SacI (**Supplementary Table 1**.), and ligated into pUCP24/T downstream of the tetracycline promoter (Gato et al., 2020; Gato, 2021). The construct (pUCP24/T_*fhaC*) was verified by PCR and introduced into Kp3380Δ*fhaC* by electroporation. Transformants were selected on gentamicin (50 µg mL^−1^).

### *In vitro* growth rates

Growth rates kinetics were assessed by measuring the optical density (OD) of the wildtype and the mutant derivatives strains in MH. Growth in 48-well plates was monitored at OD600 every 1 hour until the late-log phase using the Epoch 2 Microplate Spectrophotometer (BioTek Instruments, USA). The value of X at the inflection point of the curve (Xint) and growth rate constant (k) were calculated using the Logistic growth curve model. Three independent biological replicates were tested. Statistical analysis was performed using nonlinear regression (logistic growth model) followed by ANOVA, as well as by calculating the area under the curve (AUC).

### Adherence to human epithelial cells

Adhesion assays were performed following a modified protocol of Rumbo-Feal et al (Rumbo-Feal et al., 2017; Gato, 2021). Human colorectal epithelial HT-29 cells (ATCC^®^ HTB-38™) were cultured in McCoy’s 5A medium (Gibco) with 10% FBS, 1% penicillin/streptomycin, and 1% GlutaMAX at 37 °C, 5% CO_2_. Monolayers (~10^5^ cells/well) in 24-well plates were washed with glucose-free HBSS and infected with ~10^5^ colony-forming units (CFU) of each bacterial strain for 3 h at 37 °C. After washing, cells were lysed with sodium deoxycholate and serial dilutions were plated to enumerate adherent CFU. Percent adherence was calculated relative to the inoculum. Six or seven independent experiments were performed; data were analyzed by ANOVA with Tukey’s *post-hoc* test, using Kp3380 as the control.

### Quantification of biofilm formation

Biofilm assays were performed following the protocol of Tomaras et al. with minor modifications (Tomaras et al., 2003; Gato, 2021). Overnight LB cultures were diluted 1:100 in fresh LB and incubated statically in 48-well polystyrene plates for 24 h at 37 °C. Total growth was measured as optical density at 600 nm (OD_600_). Biofilm biomass was stained with crystal violet, solubilized in 30% acetic acid, and then quantified by measuring OD_580_. Biofilm formation was expressed as the OD_580_/OD_600_ ratio. Five or six independent replicates were analyzed by ANOVA with Tukey’s *post-hoc* test.

### *In vitro* competition assays

Isogenic pairs (Kp3380/Kp3380Δ*kpiD*, Kp3380/Kp3380Δ*fhaC* and Kp3380/Kp3380Δ*kpiD*Δ*fhaC*) were mixed 1:1 at ~0.5 McFarland in 10 mL LB (Gato, 2021). After ~20 generations (16–18 h, 37 °C, 180 rpm), cultures were serially diluted and plated on LB and LB with apramycin (200 µg mL^−1^). The competition index (CI) was calculated as (mutant CFU/wild-type CFU) final ÷ (mutant CFU/wild-type CFU) initial. Three independent replicates were realized. Means from these independent experiments were considered.

### A murine model of intestinal colonization

Intestinal colonization was evaluated using a previously described murine model (Gato et al., 2020), with some modifications. Six-week-old female BALB/c mice were used and randomly assigned to individual cages (one mouse per cage). Prior to infection, the animals were treated with a single dose of streptomycin (1 g/kg in 100 μL of water, administered by oral gavage) to eliminate the resident gut microbiota. Subsequently, the mice were individually inoculated with strains Kp3380 or Kp3380Δ*fhaC* (∼1.6-1.9 × 10^6^ CFU in 100 μL of PBS). To prepare the inoculum, 100 mL of fresh LB broth was inoculated into a 250-mL Erlenmeyer flask with 1 mL of an overnight culture, and incubated at 37 °C with shaking (180 rpm) until an OD_600_ of 0.7 was reached. The bacteria were harvested by centrifugation (4,000 × g, 15 min), washed twice, and resuspended in PBS. Three days after inoculation, the animals were euthanized, and intestinal segments (cecum and colon) were isolated under aseptic conditions. The tissues were weighed prior to homogenization and inoculated onto LB plates supplemented with kanamycin (50 mg/mL) to determine the bacterial load. All mice were maintained in the specific pathogen-free facility at the Technology Training Centre of the A Coruña University Hospital Complex (Spain).

### Ethics

Animal experiments were carried out with the approval of and in accordance with regulatory guidelines and standards established by the Animal Ethics Committee of the A Coruña University Hospital Complex (project code 15002/2020/010). This committee follows the recommendations of the National Committee for the Replacement, Refinement, and Reduction of Animal Research.

### Statistical analysis

Statistical analysis was performed using nonlinear regression (logistic growth model) followed by ANOVA, as well as by calculating the area under the curve (AUC) in growth curves. Statistical significance among groups was assessed using one-way analysis of variance (ANOVA) followed by Tukey’s *post hoc* test to compare each condition with the corresponding control in both adherence and biofilm formation assays. Data were analyzed using GraphPad Prism 10.0.0, which was also employed to generate all graphical representations.

## Results

### Computational characterization of the KpFhaB/FhaC system in *K. pneumoniae* Kp3380

**Figure 1:**
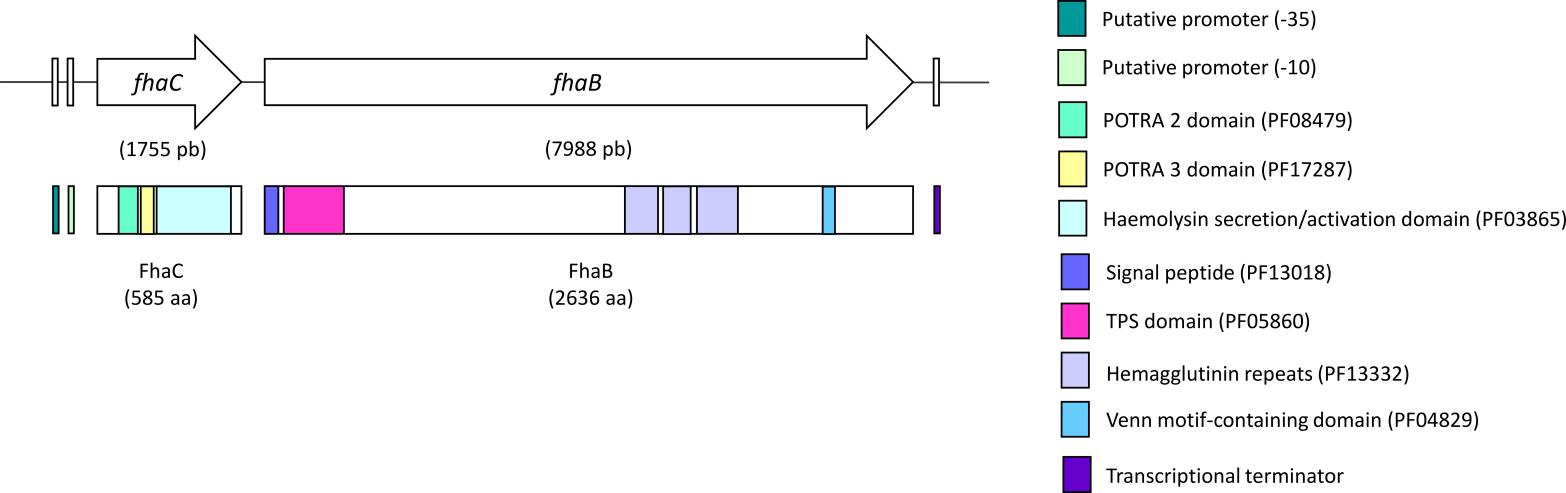
Genetic organization of the KpFhaB/FhaC TPS system in *K. pneumoniae* Kp3380 strain. Arrows indicate the location and direction of gene transcription, and colors indicate functions. Functional domains are pointed.

Whole-genome sequencing of the *K. pneumoniae* strain Kp3380 (AN2344; Bioproject PRJEB39112) revealed a 10.7 kb chromosomal region encoding two adjacent genes: AN2344V1_4287 (7,908 bp) and AN2344V1_4288 (1,755 bp). The former gene encodes a protein resembling the FhaB adhesin, while the later encodes a protein resembling the FhaC outer membrane protein (**Figure 1**). These proteins were therefore identified as the components of a TPS system, hereafter designated as KpFhaB/FhaC.

Protein homology and domain bioinformatic tools predicted that KpFhaB encoded a TpsA-family exoprotein with a 49-amino acid signal peptide (Pfam Id: PF13018). The protein sequence harbors a conserved TPS domain (residues 69–330; Id: PF05860), a transport domain near the N-terminus, and a Venn motif-containing domain (residues 2369–2417; Id: PF04829), commonly found in bacterial toxins near the C-terminus. Additionally, three hemagglutinin repeats (associated with host-cell binding and infection; Id: PF13332) were also identified in the C-terminal half of the protein. (**Figure 1**).

KpFhaC was predicted to encode a TpsB-like outer membrane protein with a 20-mer signal peptide. The protein contains two POTRA domains (POTRA 2, Id: PF08479, residues 99–175; POTRA 3, Id: PF17287, residues 177–230) implicated in protein transport and chaperone-like activity, and a hemolysin secretion/activation domain (Id: PF03865, residues 235–548) essential for TpsA exoprotein translocation (**Figure 1**). These functional features suggest that KpFhaC mediates the secretion and activation of KpFhaB as part of the TPS system.

### KpFhaB/FhaC is strongly associated with the high-risk clone *K. pneumoniae* ST15

**Figure 2:**
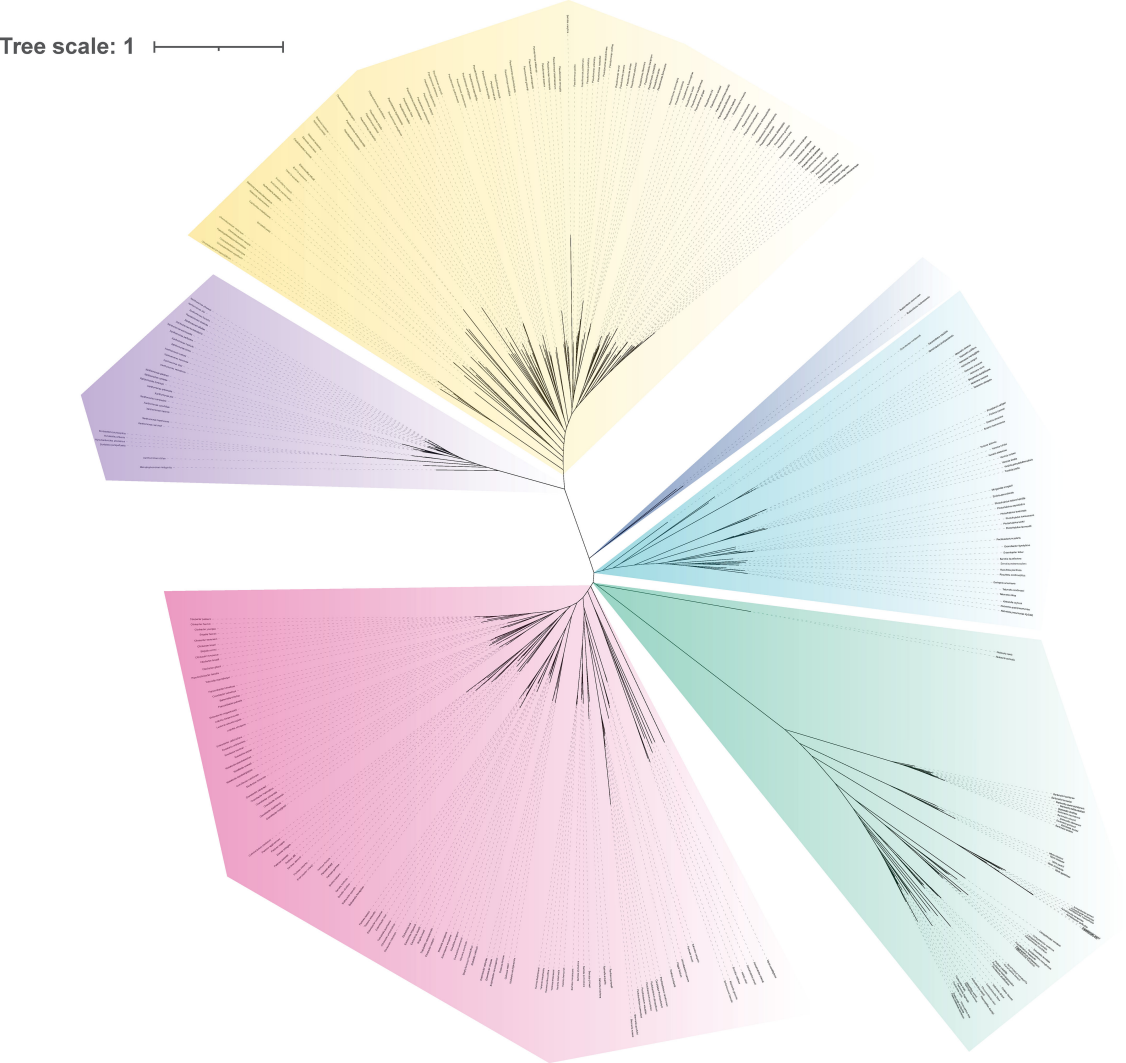
Maximum-likelihood (ML) phylogenetic tree of the Kp3380 *fhaB* gene in comparison with KpFhaB/FhaC-related sequences retrieved from the BV-BRC database. Each color represents a clade. Graphic representation was generated using iTOLv7.

Phylogenetic analysis of the Kp3380 *fhaB* gene against homologs from the BV-BRC database revealed six clades. The Kp3380 *fhaB* gene clustered with other from 43 species from six taxonomic families: *Enterobacteriaceae*, *Yersiniaceae*, *Pectobacteriaceae*, *Morganellaceae*, and *Erwiniaceae* (**Figure 2**, blue clade). The closest relatives included *Klebsiella quasipneumoniae* and *Klebsiella oxytoca*, followed by *Tatumella citrea*, *Tatumella morbirosei*, and *Ewingella americana*.

**Figure 3:**
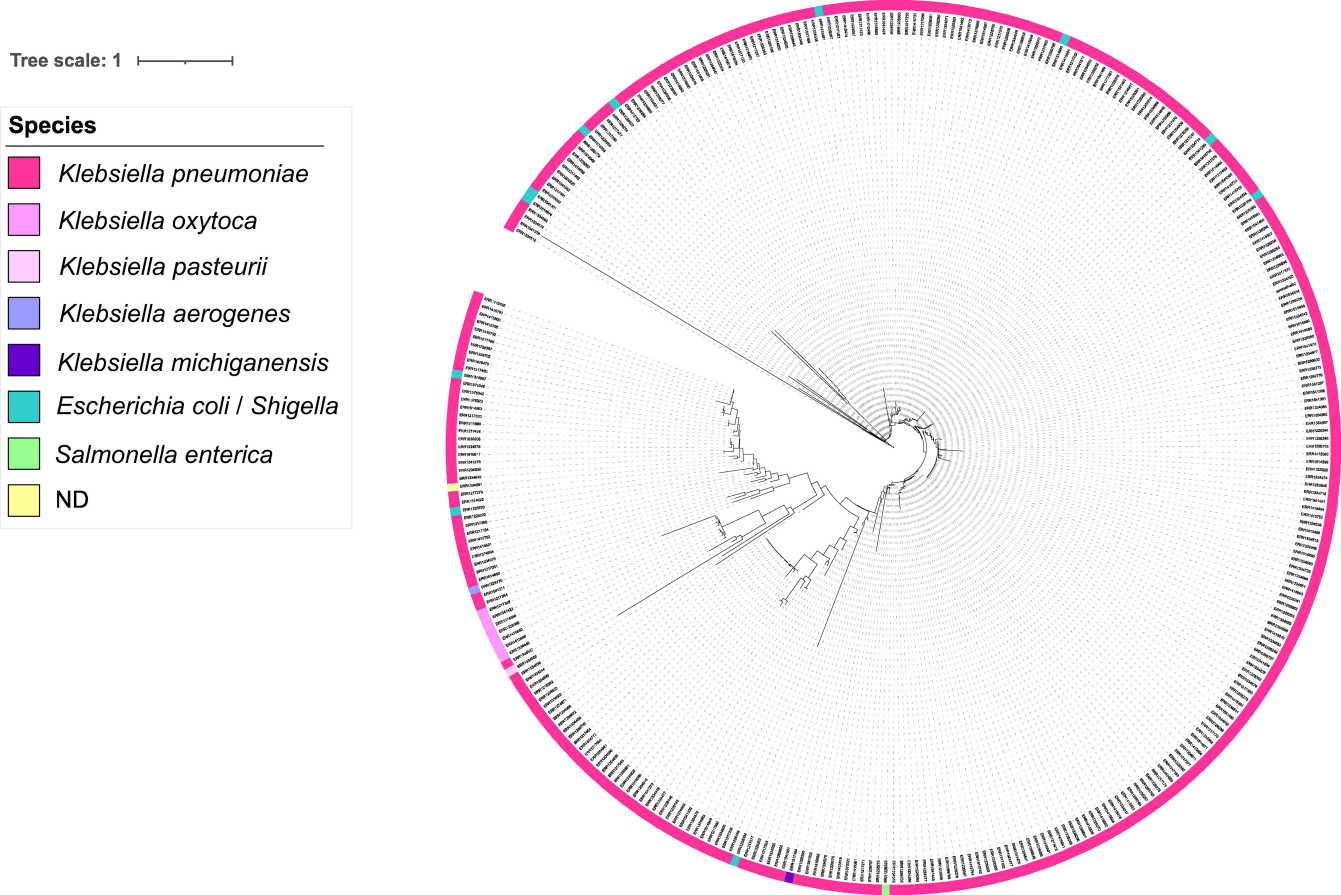
Maximum-likelihood tree obtained by comparing the *fhaB* and *fhaC* gene sequences of strain Kp3380 with those detected in the EuSCAPE project collection. The ideogram includes species information, and the tree was visualized using iTOLv7.

Moreover, *fhaB* and *fhaC* were identified in 334 Enterobacteriaceae isolates included in the EuSCAPE project. The Kp3380-like TPS was most commonly found in *K. pneumoniae*, but it was also detected in other species, including *E. coli/Shigella*, *Klebsiella aerogenes*, *Klebsiella michiganensis*, *Salmonella enterica*, and one undetermined species (**Figure 3**).

**Figure 4:**
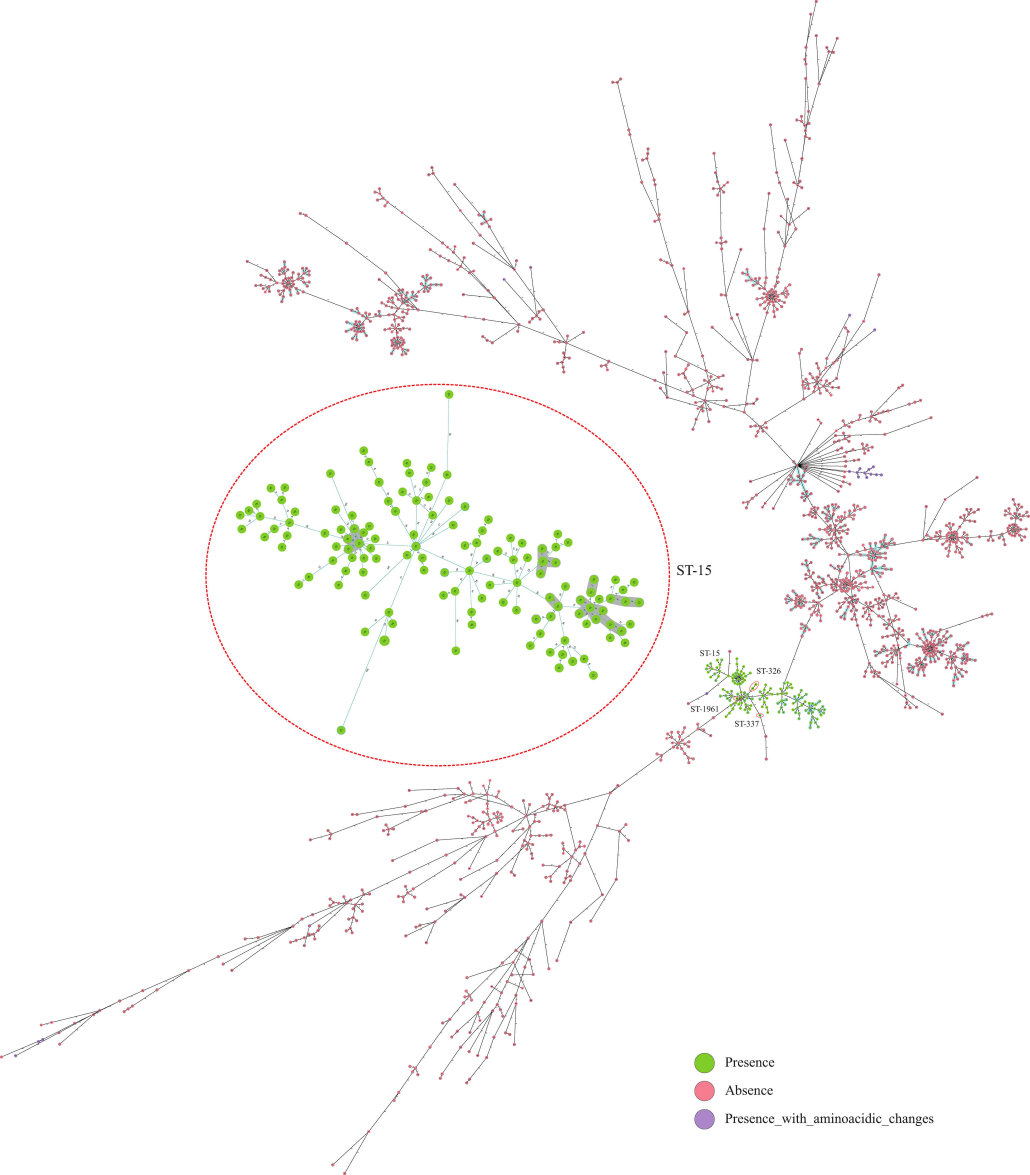
Population structure of 1,649 *K. pneumoniae* isolates collected during the EuSCAPE survey. Minimum-spanning tree based on cgMLST of 2,358 genes (pairwise ignoring missing values). Node colors indicate the status of the *fhaC* gene: presence (green), absence (red), or presence with amino acid changes (purple). Labels indicate the sequence type (ST) for specific isolates. The dached red oval highlights the ST15 cluster. Grey and blue shadows represent strain groups defined by a threshold of 5 alleles.

Strikingly, the *fhaC* gene was detected in only 10.25% (169/1,649) of *K. pneumoniae* isolates from the EuSCAPE collection. The majority of these (85.8%, 145/169) belonged to ST15 and shared 100% amino acid identity with Kp3380 (**Figure 4**). Among the remaining STs, only ST326 (2 isolates), ST337 (1 isolate), and ST1961 (1 isolate) were identical to Kp3380. Other isolates showed few amino acid variations (**Figure 4**).

### The *fhaBC* genes belongs to a predicted genomic cluster probably acquired from *Klebsiella variicola*

All non-ST15 *K. pneumoniae* isolates carrying the KpFhaB/FhaC system showed varying degrees of relatedness, single- to tetra-locus variants in the MLST scheme, to ST15. However, all isolates belonging to the single-locus variant ST14, another intrahospital lineage phylogenetically related to ST15 (Wyres et al., 2020), lacked KpFhaB/FhaC. No additional *fhaBC*-positive isolates were identified among a dataset of 2,056 complete *Klebsiella* genomes, comprising 296 distinct STs retrieved from the RefSeq database (May 6, 2024).

Transcriptional elements were searched in non-coding regions flanking the cluster genes. Upstream of the first gene in the cluster, two potential promoter elements containing recognizable −35 and −10 boxes were detected ([ATGAAA–19 nt–TATAAT] located 121 nt upstream of the start codon; [TTGATA–20 nt–TATTAA] located 53 nt upstream). A transcriptional terminator was predicted downstream the last gene. Overall, these features are consistent with all genes in the cluster are co-transcribed as an operon (**Figure 1**).

Because the cluster was confined to a small subset of *K. pneumoniae* STs, we investigated potential signatures of mobile genetic elements (MGE) involving inter-species gene horizontal transfer. The *fhaBC* cluster displayed an A+T content of 43.3%, closely matching the 42.3% median (Z-score 0.223, *P* = 0.588) of the *K. pneumoniae* core genome (https://www.cgmlst.org). Likewise, the correspondence analysis of codon usage for *fhaB* and *fhaC* showed no deviation from the genomic background (**Supplementary Figure 1**). The cluster was consistently detected in a single chromosomal context, with no evidence of prophages, transposons, insertion sequences, direct repeats, or plasmid association, suggesting that it is not part of an active mobile element. A whole-cluster BLAST search revealed top hits to *Klebsiella variicola*, with up to 95% sequence coverage and 98% nucleotide identity. Remarkably, over half of the analyzed *K. variicola* isolates harbored a cluster homologous to *K. pneumoniae fhaBC*.

Altogether, these findings strongly support the horizontal acquisition of the KpFhaB/FhaC system from *K. variicola*, followed by long-term chromosomal integration and compositional adaptation to translational machinery within the *Klebsiella* genus.

### Growth kinetics of Kp3380 are unaffected by *fhaC* and *kpiD* inactivation

**Supplementary Figure 2** shows the growth curves of all the strains of interest evaluated in this study. To confirm that the differences observed in adherence and biofilm formation were not due to an underlying growth defect, the growth kinetics of the Kp3380 strain and its derivative mutants were analyzed. The Kp3380Δ*kpiD* and Kp3380Δ*fhaC* mutants showed a growth curve almost identical to that of the wildtype strain Kp3380. However, the Kp3380Δ*kpiD*Δ*fhaC* strain showed significant differences relative to Kp3380 strain during the early stationary phase, stabilizing toward the end of this phase. The Kp3380Δ*kpiD*_C and Kp3380Δ*fhaC*_C strains showed differences in the exponential phase compared to wildtype strain, stabilizing at the end of the stationary phase (**Supplementary Figure 2**, **Supplementary Table 2**.).

### Effect of *fhaC* deletion in adherence to human cells

**Figure 5:**
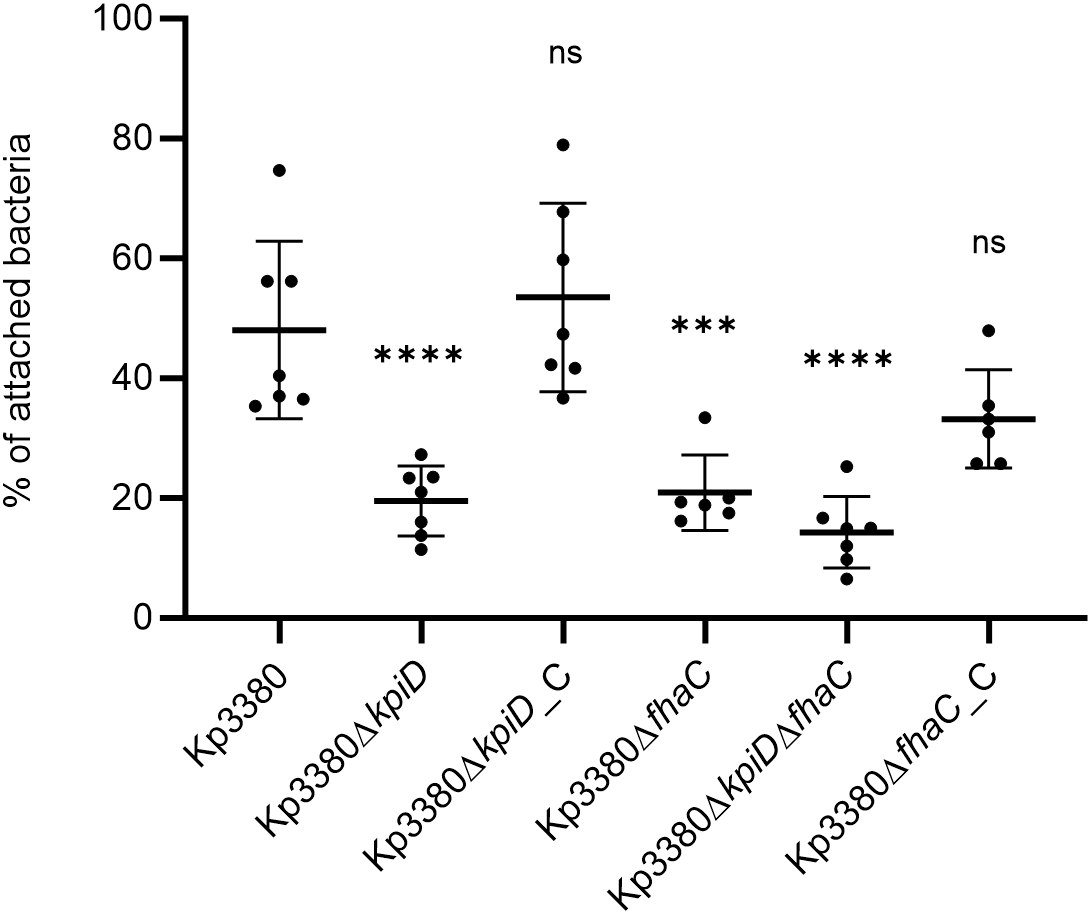
Quantification of the adherence of *K. pneumoniae* strains to HT-29 intestinal colorectal cells. Bacterial adherence is reported as the percentage of attached bacteria relative to the initial inoculum (100%). Kp3380 is the wild-type strain, while its isogenic derivatives include mutants Kp3380Δ*fhaC*, Kp3380Δ*kpiD*, the double mutant Kp3380Δ*kpiD*Δ*fhaC*, and complemented strains Kp3380Δ*fhaC_C* and Kp3380Δ*kpiD_C*. Each symbol represents an individual biological replicate; the wild-type and single mutants were tested in 7 independent replicates (n=7), whereas the double mutant and complemented strains were tested in 6 replicates (n=6). Horizontal lines indicate the mean value for each group and error bars represent the standard deviation (SD). Statistical significance was determined by one-way ANOVA followed by a *post hoc* Tukey’s multiple comparisons test to identify differences between each derivative and the wild-type control. Significance levels are indicated as follows: non-significant (ns), *** P ≤ 0.001, and **** P≤ 0.0001.

To investigate the contribution of the *K. pneumoniae* TPS system KpFhaB/FhaC to bacterial adherence, we quantified the interaction of the wild-type strain Kp3380 and its isogenic mutants with HT-29 human colorectal epithelial cells. This cell line was chosen to specifically model the intestinal niche, given that both KpFhaB/FhaC and the KpiD fimbrial system are co-selected in the ST15 lineage, suggesting a specialized role in GI tract colonization. Under these conditions, deletion of *fhaC* alone (Kp3380Δ*fhaC*) led to a significant 52.7% reduction in adherence (**Figure 5**) compared to the wild-type strain (*P* < 0.0001, Student’s t-test) (Gato, 2021).

Given the previously established contribution to this function of *kpiD*, part of another adherence-related system also associated with ST15, we next examined a double mutant lacking both *kpiD* and *fhaC* (Kp3380Δ*kpiD*Δ*fhaC*). This strain exhibited an even more pronounced 71.4% decrease in adherence relative to the wild-type (*P* < 0.0001), suggesting an additive effect of disrupting both systems. A previously characterized *kpiD* mutant (Kp3380Δ*kpiD*) (Gato et al., 2020) was included in the assay as a control, confirming its known role in adherence reduction under the tested conditions.

### Deletion of *fhaC* reduce biofilm formation

**Figure 6:**
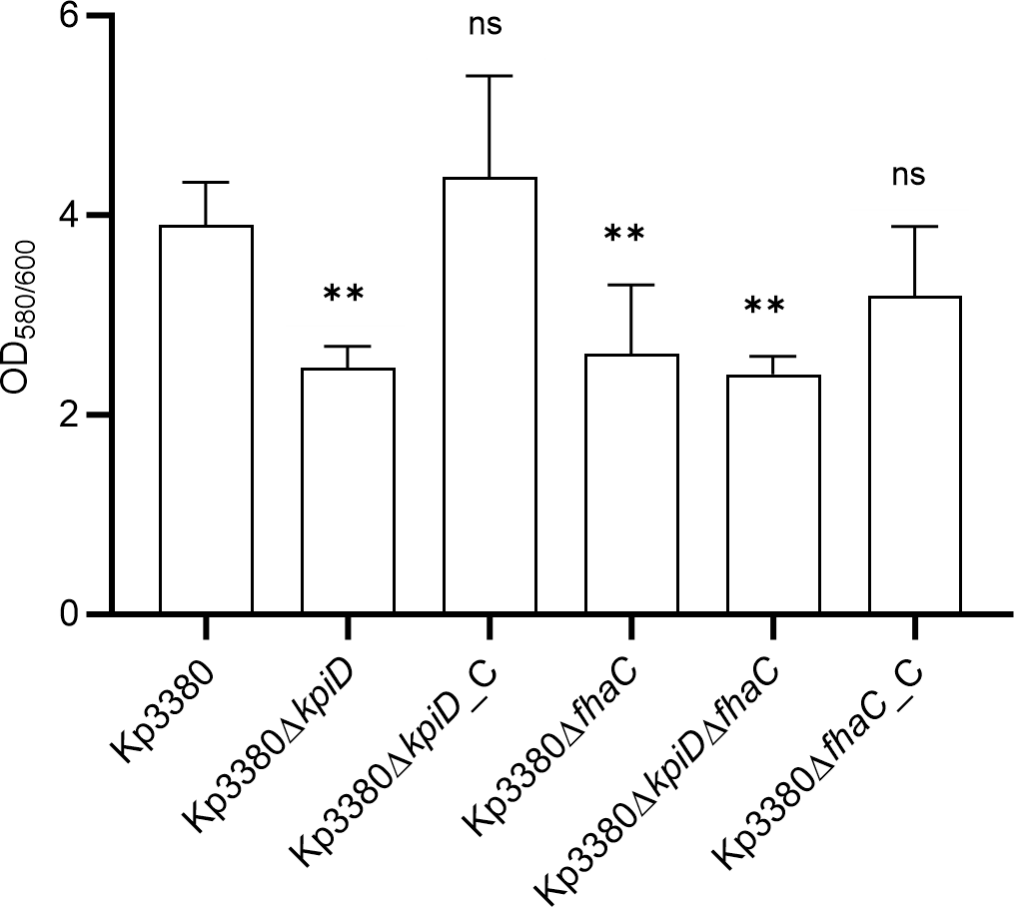
Quantitative analysis of biofilm formation by *K. pneumoniae* isolates. Biofilms produced by the wild-type strain (Kp3380) and its derivatives, including mutants Kp3380Δ*fhaC*, Kp3380Δ*kpiD*, and Kp3380Δ*kpiD*Δ*fhaC*, as well as complemented strains Kp3380Δ*fhaC*_C and Kp3380Δ*kpiD*_C, were quantified by crystal violet staining. Each symbol represents an individual biological replicate; the wild-type and single mutants were tested in six replicates (n=6), while the double mutant and complemented strains were tested in five replicates (n=5). Horizontal lines indicate the mean value for each group, and error bars represent the standard deviation (SD). ANOVA was used to detect any significant differences between the groups, and a *post hoc* Tukey’s test was used to determine the difference between each group and the respective control (Kp3380). Statistical significance levels are indicated as non-significant (ns) and ** P ≤ 0.01).

Biofilm formation assays revealed that inactivation of the KpFhaB/FhaC secretion system negatively affected biofilm formation in the strain Kp3380. The Kp3380Δ*fhaC* mutant showed a 34.2% reduction in biofilm formation (**Figure 6**) compared to the wild-type strain (*P* < 0.05). The Kp3380Δ*kpiD* mutant, previously characterized for its role in adherence and included here as a control, showed a similar impaired biofilm formation under the same conditions (**Figure 6**) (Gato, 2021).

The double mutant lacking both *kpiD* and *fhaC* (Kp3380Δ*kpiD*Δ*fhaC*) exhibited a 41.2% reduction in biofilm formation (**Figure 6**) relative to the wild-type (P < 0.01). Complementation of each mutant restored biofilm formation to wild-type levels.

### Fitness cost associated with inactivation of *fhaC* gene

**Figure 7:**
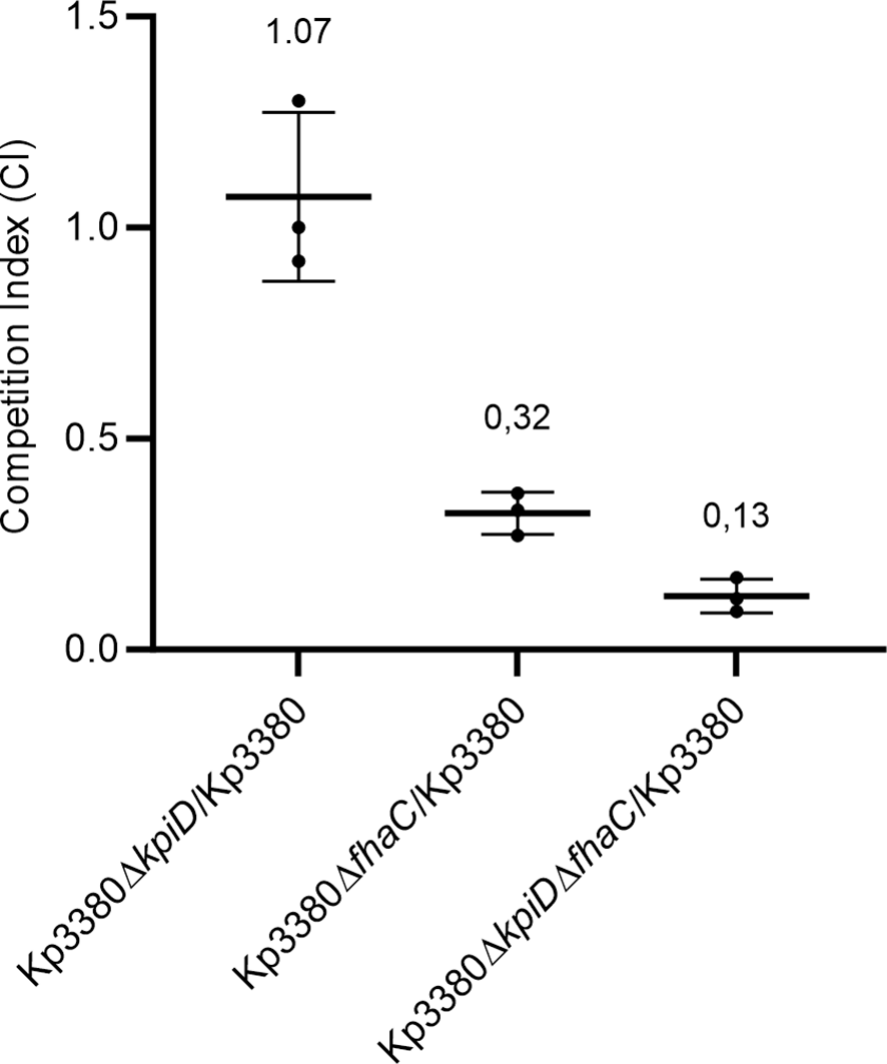
*In vitro* competition assays between K. pneumoniae Kp3380 and its isogenic mutants. Competitive experiments were performed for the pairs Kp3380/Kp3380Δ*kpiD*, Kp3380/Kp3380Δ*fhaC* and Kp3380/Kp3380Δ*KpiD*Δ*fhaC*. The competition index (CI) was calculated as the ratio of mutant to wild-tupe CFU at the final timepoint, normalized by the initial ratio in the inoculum: CI= (mutant CFU/wild-type CFU) final/(mutant CFU/wild-type CFU) initial. Each symbol represents an individual biological replicate (n=3). Horizontal lines indicate the mean value for each group, and error bars represent the standard deviation (SD). A CI value significantly lower than 1 indicates a competitive disadvantage of the mutant strain.

*In vitro* competition assays were performed to evaluate the fitness cost associated with inactivation of the KpI and KpFhaB/FhaC systems in the strain Kp3380. All mutant strains displayed a significantly reduced competitive ability. The Kp3380Δ*kpiD* mutant showed an average CI value of 1.07 relative to the wild type, the Kp3380Δ*fhaC* a mean CI of 0.32 and the Kp3380Δ*kpiD*Δ*fhaC* double mutant exhibited a further reduction in fitness, with a mean CI of 0.13 compared to the wild-type strain, indicating a substantial fitness cost associated with the inactivation of both systems(**Figure 7**) (Gato, 2021).

### Role of the KpFhaB/FhaC secretion system in intestinal colonization

**Figure 8:**
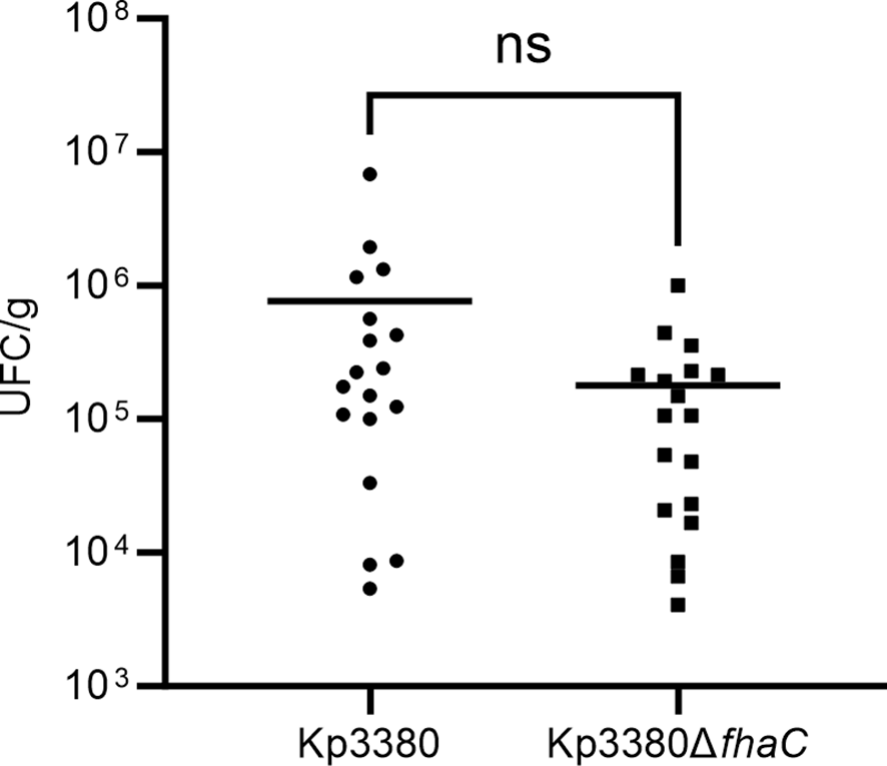
Intestinal colonization of mice with the Kp3380 parental strain or the Kp3380Δ*fhaC* mutant was achieved by oral gavage with ∼1.6-1.9 x 10^6^ bacteria. The bacterial burden in intestinal segments (cecum and colon) was determined 72 h post-inoculation. Each symbol represents an individual biological replicate (n=18). Horizontal lines indicate the mean value for each group, and error bars represent the standard deviation (SD). The Mann–Whitney test was used to determine significant differences between the two groups. Statistical significance levels are indicated as non-significant (ns).

Given the role of the KpFhaB/FhaC secretion system in *in vitro* adhesion to HT-29 colorectal cells, *in vivo* murine models were used to evaluate its involvement in intestinal colonization. Specifically, the colonization ability of the Kp3380Δ*fhaC* strain was analyzed. Mice that had been pre-treated with streptomycin (18 per group) were individually inoculated orally with the parental strain Kp3380 (1.6 × 10^6^ CFU in 100 μL of PBS) or with the mutant strain Kp3380Δ*fhaC* (1.9 × 10^6^ CFU in 100 μL of PBS). Three days after inoculation, the animals were euthanized to determine the bacterial load in the intestine. A trend toward slightly lower bacterial levels was observed in the cecum and colon of mice colonized with the mutant strain compared to those colonized with the wild-type strain; however, these differences were not statistically significant (**Figure 8**). As a negative control, mice orally inoculated with PBS were included; no bacteria were detected in the intestinal segments analyzed.

## Discussion

The contribution of TPS systems to adherence, biofilm formation, and host-cell interactions is well known in Gram-negative pathogens (Van Ulsen et al., 2014; Pérez et al., 2017; Meuskens et al., 2019). While TPS systems have been extensively characterized in organisms such as *B. pertussis*, *A. baumannii*, *Haemophilus influenzae* and *Erwinia chrysanthemi* (Rojas et al., 2002; St. Geme and Yeo, 2009; Melvin et al., 2015; Rempe et al., 2016; Pérez et al., 2017), their evolutionary acquisition and functions in high-risk *K. pneumoniae* clones remain largely unexplored. In this study, we characterized for the first time the role of the FhaB/FhaC system in adherence to intestinal epithelial cells, biofilm formation, and bacterial fitness in *K. pneumoniae*. The KpFhaB protein shares most of its protein domains with other TPS adhesins. When compared with FhaB from *Acinetobacter baumannii* (AbFhaB), KpFhaB contains a C-terminal pre-toxin domain that is absent in AbFhaB, which instead contains a Colicin D-like domain which is functionally analogous in mediating host interactions (Pérez et al., 2017). The POTRA domains (POTRA_2 and POTRA_3), which are likely involved in the recognition and translocation of the adhesin across the outer membrane or its integration into it, are shared in the KpFhaC and AbFhaC proteins (Clantin et al., 2004; Thanassi et al., 2004). Diverse Gram-negative bacteria share these structural features suggesting a conserved role for TPS in adhesion. This idea is also supported by our phylogenetic analysis, which showed that this system is also evolutionarily conserved among *Enterobacteriaceae* and related families. Previous studies have shown that *fha*-like genes are widespread among diverse pathogens like FHA in *B. pertussis*, HMW1/HMW2 in *H. influenzae*, EtpA/EtpB in *E. coli* or CdrA in *Pseudomonas aeruginosa*, where they act as key adhesion and virulence factors (Melvin et al., 2015; Guérin et al., 2017).

We analyzed the presence of KpFhaBC in a representative collection of hospital-acquired *K. pneumoniae* in Europe (David et al., 2019) and observed that this TPS system was even more closely associated with ST15 than the previously described adherence-related Kpi system (Gato et al., 2020). Therefore, we sought to investigate the evolutionary pathway of this system in *K. pneumoniae.* Phylogenetic analysis revealed that the *K. pneumoniae* Kp3380 TPS system has an unusual distribution, clustering with both closely related species such as *K. quasipneumoniae* and *K. oxytoca* and more distant such as *T. citrea*, *T. morbirosei* or *E. americana*. This broad distribution contradicts the pattern expected from vertical inheritance from a *K. pneumoniae* ancestor, instead showing a trajectory from external acquisition, through horizontal gene transfer, to clade-specific conservation. The high degree of conservation of KpFhaB/FhaC among ST15 isolates, contrasted with its absence in most other lineages, raises questions about its acquisition and the selective pressures driving its maintenance within this high-risk clone.

The absence of canonical MGE signatures, together with the highly conserved genomic context and the close genetic relatedness of all KpFhaB/FhaC-carrying isolates, strongly suggests that the TPS system KpFhaB/FhaC was introduced into the *K. pneumoniae* lineage through a single horizontal transfer event. We identified *K. variicola* as the most probable donor, given the higher prevalence of this TPS cluster in its genome and the similar ecological niche of both species in the human gastrointestinal tract, conditions that may facilitate rare interspecies genetic transfer. The A+T content and codon usage patterns of the cluster, which are consistent with the rest of the *Klebsiella* genome, suggest subsequent adaptation and stable integration. We propose that the transfer likely occurred via the uptake of linear DNA fragments, a low-efficiency but feasible process in natural environments, leading to the stable chromosomal anchoring of this adhesion operon without the typical characteristics of a mobilizable element (Navon-Venezia et al., 2017). As previously observed in the TPS systems of *A. baumannii* and *B. pertussis*, the presence of *fhaB* in certain lineages of *K. pneumoniae* could confer an adaptive advantage due to its ability to promote epithelial adhesion or biofilm development (Serra et al., 2011; Pérez et al., 2017). The conservation of the KpFhaB/FhaC system in specific *K. pneumoniae* clones is not random, but could be a consequence of sustained adaptive pressures. This selective advantage is likely due to the operon’s contribution to key pathogenic traits, such as adaptation to ecological niches or virulence, which could facilitate persistence and dissemination in healthcare settings.

Adherence is a critical pathogenic trait, facilitating initial bacterial attachment to host tissues, colonization, and biofilm formation, which in turn promotes evasion of host defenses and long-term persistence (Paczosa and Mecsas, 2016; Martin and Bachman, 2018). TPS systems are well known virulence factors that mediate bacterial adherence to host cells and surfaces. This adhesion facilitates colonization and biofilm formation in diverse pathogens, including *Bordetella bronchiseptica* (Inatsuka et al., 2005a), *Neisseria meningitidis* (Schmitt et al., 2007), *Haemophilus ducreyi* (Ward et al., 2003) and enterotoxigenic *E. coli* (Roy et al., 2008).

In this work, we demonstrated that functional inactivation of the FhaB/FhaC system in the Kp3380 strain significantly reduced adhesion to both biotic and abiotic surfaces. Our results are in agreement with those found in *B. pertussis* and *A. baumanii*, among others (Ward et al., 2003; Inatsuka et al., 2005b; Schmitt et al., 2007; Roy et al., 2008; Serra et al., 2011; Darwish Alipour Astaneh et al., 2017). Furthermore, the ability of Kp3380 to attach to HT-29 colorectal epithelial cells was significantly reduced when FhaBC system was inactivated, which point out its contribution to host-cell interactions. This adherent phenotype was even more impaired when both adherence-related systems, Kpi and FhaBC, were inactivated, suggesting a cooperative relationship between these TPS and CUP systems. Similar findings regarding the additive contribution of different adhesion-related systems were described in other pathogens such as *Bordetella* species (Relman et al., 1989; Serra et al., 2011; Scheller et al., 2015).

Considering the role of KpFhaB/FhaC and Kpi systems in the adherent phenotype (Gato et al., 2020) of *K. pneumoniae* ST15, we hypothesize that this clone may have selectively conserved both adhesion mechanisms as an adaptive advantage that contributes to its epidemic success.

Our competition assays suggest that the KpFhaB/FhaC system plays a significant role in bacterial competition, whereas Kpi contributes additively or synergistically in the absence of *fhaC*. Consistent with this, analysis of the growth curves revealed limited variations in parameters such as k, Xint, and AUC among the different strains. This indicates that the observed differences in fitness are not due to global growth defects, but rather to finer alterations in relative fitness that manifest specifically under co-culture conditions. Interestingly, although *fhaC* appears to be a major determinant of competitive fitness *in vitro*, its role in intestinal colonization *in vivo* was not significant in our murine model. The absence of a clear phenotype for the *fhaC* mutant *in vivo* could be explained by functional compensation provided by the Kpi system, which has been shown to be essential for colonization, as previously described (Gato et al., 2020), although it does not independently affect competitive fitness.

These divergent results suggest functional specialization and synergy between the KpFhaB/FhaC and Kpi systems, which could underpin the evolutionary success of the ST15 lineage. It appears that ST15 utilizes these systems to meet different ecological requirements: the KpFhaB/FhaC system likely facilitates survival in the environment and the displacement of competing microbiota, while Kpi ensures stable adhesion to the host. The additive effect observed in the fitness of the double mutant Kp3380Δ*kpiD*Δ*fhaC* suggests that Kpi provides a compensatory advantage when *fhaC* is absent.

From a clinical perspective, the coexistence of these specialized adhesion mechanisms could be key to persistence in healthcare settings. The ability to compete with neighboring strains via the KpFhaB/FhaC system and to maintain robust colonization through Kpi likely enhances ST15’s capacity to colonize patients, withstand environmental pressures on hospital surfaces, and ultimately facilitate long-term transmission. This strategy highlights how the integration of diverse colonization factors allows high-risk clones to adapt flexibly to the changing niches of the hospital environment.

## Data Availability

The datasets presented in this study can be found in online repositories. The names of the repository/repositories and accession number(s) can be found in the article/**Supplementary Material**. The data are also available in Figshare under the following DOI: https://doi.org/10.6084/m9.figshare.30739031.
