# Correction: KpFhaB/FhaC is a virulence-associated TPS system of the globally-disseminated *Klebsiella pneumoniae* ST15 high-risk clone

**DOI:** 10.3389/fcimb.2026.1911734

**Published:** 2026-07-20

**Authors:** Ana Tajuelo, Eva Gato, Carlota Moya, Sonia Prieto Martín-Gil, Beatriz Cano-Castaño, Leilani Vaughan, Pedro Miguela-Villoldo, María Pérez-Vázquez, Miriam Moscoso, Bruno Kotska Rodiño-Janeiro, Félix Docando, María C. Terrón, Antonio J. Martín-Galiano, Michael J. McConnell, Germán Bou, Astrid Pérez

**Affiliations:** 1 Laboratorio de Infecciones Intrahospitalarias, Centro Nacional de Microbiología, Instituto de Salud Carlos III (ISCIII), Madrid, Spain; 2 Escuela internacional de Doctorado, Ciencias de la Salud, Universidad Nacional de Educación a Distancia (UNED), Madrid, Spain; 3 Servicio de Microbiología, Hospital Universitario A Coruña, Instituto de Investigación Biomédica de A Coruña (INIBIC), Universidad de A Coruña, A Coruña, Spain; 4 Laboratorio de Referencia e Investigación en Resistencia a Antibióticos e Infecciones Relacionadas con la Asistencia Sanitaria, Centro Nacional de Microbiología, Instituto de Salud Carlos III, Madrid, Spain; 5 Centro de Investigación Biomédica en Red de Enfermedades Infecciosas (CIBERINFEC), Instituto de Salud Carlos III, Madrid, Spain; 6 Electron Microscopy Unit, Central Core Facilities, Instituto de Salud Carlos III, Majadahonda, Madrid, Spain; 7 Unidad de Proteómica, Unidades Centrales Científico-Técnicas, Instituto de Salud Carlos III (ISCIII), Madrid, Spain; 8 Department of Biological Sciences, University of Notre Dame, Notre Dame, IN, United States; 9 Eck Institute for Global Health, University of Notre Dame, Notre, Dame, IN, United States; 10 Departamento de Fisioterapia, Medicina y Ciencias Biomédicas, Universidad de A Coruña, A Coruña, Spain

**Keywords:** adherence, biofilm formation, FhaB/FhaC, *Klebsiella pneumoniae*, phylogeny, ST15

Author “Sonia Prieto Martín-Gil” was erroneously spelled as “S.P”.

Author “Pedro Miguela-Villoldo” was omitted as an author in the published article. The correct author list reads:

“Ana Tajuelo^1,2+^, Eva Gato^1,3+^*, Carlota Moya^1^, Sonia Prieto Martín-Gil^1,2^, Beatriz Cano-Castaño^1,2^; Leilani Vaughan^1^, Pedro Miguela-Villoldo^1^, María Pérez-Vázquez^4,5^, Miriam Moscoso^3,5^, Bruno Kotska Rodiño-Janeiro^3^, Félix Docando^6^, María C. Terrón^6^, Antonio J. Martín-Galiano^7^, Michael J. McConnell^8,9^, Germán Bou^3,5,10^, Astrid Pérez^1,3^*”

The **Author Contributions** Statement has been corrected with the contributions of Pedro Miguela-Villoldo, the initials of Sonia Prieto Martín-Gil have also been corrected:

“AT: Methodology, Formal analysis, Writing – original draft, Writing – review & editing. EG: Conceptualization, Methodology, Supervision, Formal analysis, Writing – original draft, Writing – review & editing. CM: Methodology, Formal analysis, Writing – original draft, Writing – review & editing. SPM-G: Methodology, Formal analysis, Writing – original draft, Writing – review & editing. BC-C: Methodology, Formal analysis, Writing – original draft, Writing – review & editing. LV: Methodology, Formal analysis, Writing – original draft, Writing – review & editing. PM-V: Methodology, Formal analysis, Writing – original draft, Writing – review & editing. MP-V: Methodology, Formal analysis, Writing – original draft, Writing – review & editing. MM: Methodology, Formal analysis, Writing – original draft, Writing – review & editing. BKR-J: Methodology, Formal analysis, Writing – original draft, Writing – review & editing. FD: Methodology, Writing – review & editing. MT: Methodology, Writing – review & editing. AJM-G: Methodology, Formal analysis, Writing – original draft, Writing – review & editing. MJM: Formal analysis, Writing – original draft, Writing – review & editing. GB: Conceptualization, Supervision, Formal analysis, Writing – original draft, Writing – review & editing. AP: Conceptualization, Supervision, Formal analysis, Writing – original draft, Writing – review & editing. Funding acquisition.”

The original version of this article has been updated.

