# Supplementary material for: KpFhaB/FhaC is a virulence-associated TPS system of the globally-disseminated Klebsiella pneumoniae ST15 high-risk clone
Source: Front Cell Infect Microbiol. 2026 Jun 3;16:1787622. doi: 10.3389/fcimb.2026.1787622 (PMC13272123; doi:10.3389/fcimb.2026.1787622)
Supplement: Supplementary file 1 [file DataSheet1.docx]

Supplementary Material

# Figure S1.

# Figure S2.


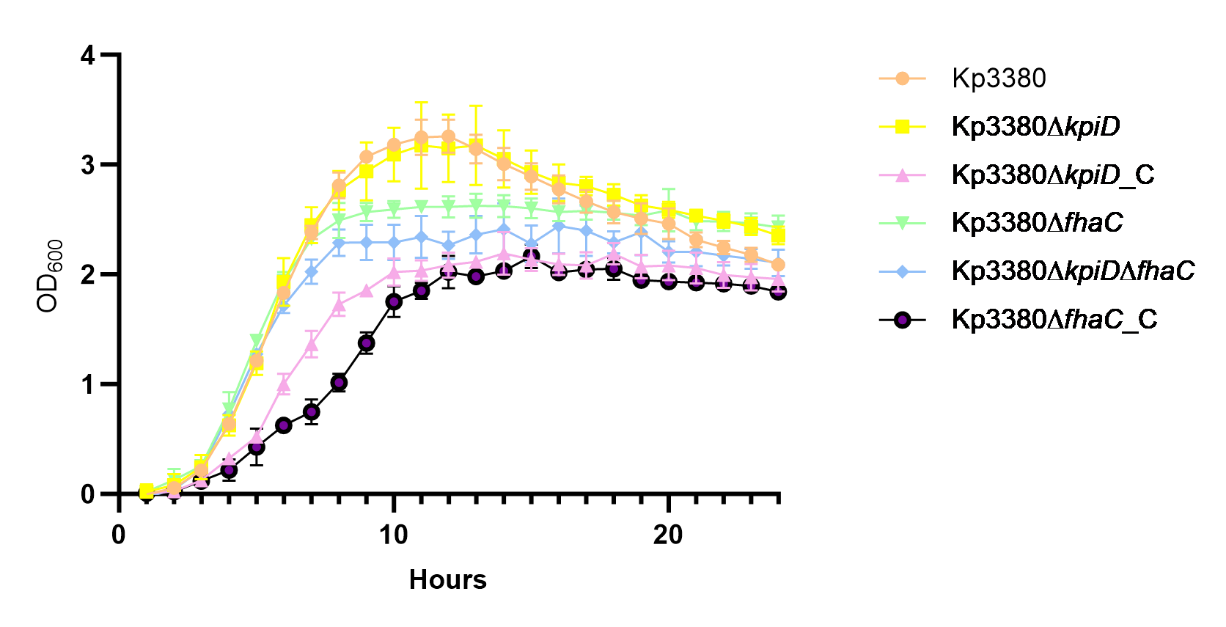


**
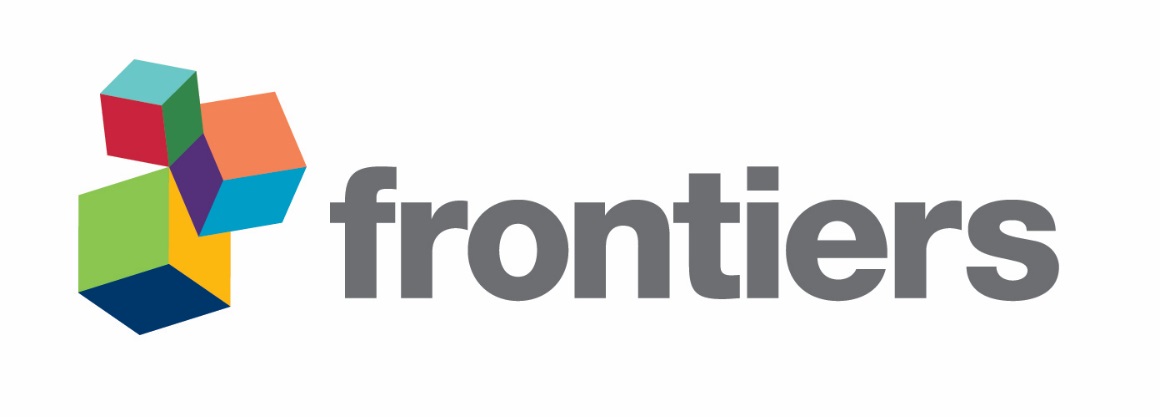
**

**Supplementary Figure 1.** AT content and codon usage of Kp-*fhaBC* genes. (A) %AT distribution of core *K. pneumoniae* genes. Values corresponding to the median of the *K. pneumoniae* core genome (green) and the *fhaBC* cluster (red) are indicated. (B) Correspondence analysis of codon usage. PC1 and PC2 values for all *K. pneumoniae* genes are shown. Location of *fhaB* and *fhaC* is highlighted in red.

**Supplementary Figure 2.** Growth curves of Kp3380 and derivative strains. The growth curves of the parental strain Kp3380 and its derivative strains are shown. Each strain is represented by a different color, as indicated in the figure legend. The data are presented as the mean ± standard deviation of three biological replicates per data point. Statistical analysis was performed using nonlinear regression (logistic growth model) followed by ANOVA, as well as by calculating the area under the curve (AUC) (Table S2.).

**Table S1.** Oligonucleotides used in the present study.

| **Primer name** | **Sequence** | **Use in the present study** |
| --- | --- | --- |
| fhac_up_fow4 | cgccgtcaccggatattcat | Amplification of the *fhaC* UP region and testing the *fhaC* gene deletion |
| fhac_up_rev4 | gcgaggctggcgggaacttcgaagttcctatactttctagagaataggaacttcgaactgcaggtcgacggatccccggaattcatcgctgtcctgcgtaag | Amplification of the *fhaC* UP region |
| fhac_down_fow1 | agtcgattggctgagctcataagttcctattccgaagttcctattctctagaaagtataggaacttcgaagcagctccagcctacacaaccgccctcagcaaaatc | Amplification of the *fhaC* DOWN region |
| fhac_down_rev1 | gctaaagggataccggcgaa | Amplification of the *fhaC* DOWN region and testing the *fhaC* gene deletion |
| fhac_apra_fow4 | gcgcctggtggtgatgccgggttatatccgtgaagccaggcttacgcaggacagcgatgaattccggggatccgtcgacc | Amplification of apramycin resistance cassette |
| fhac_apra_rev1 | ccagggcaaaaggcaaattgagctgagcattgaattgcaggattttgctgagggcggttgtgtaggctggagctgcttc | Amplification of apramycin resistance cassette |
| fhaC_Compl_SmaI_Fow | tcccccgggggaatgggtttggttgtattaaatg | Cloning of *fhaC* gene into the  pUCP24/T plasmid for complementation |
| fhaC_Compl_SacI_Rev | cgagctcgtcagtagctccagctgacgc | Cloning of *fhaC* gene into the  pUCP24/T plasmid for complementation |
| pUCp24_check_Fw | gcctgcctttcaggctgcgcaactgtt | Testing of the plasmid construction pUCP24/T_ fhaC for complementation |
| pUCp24_check_Rv | tggcacgacaggtttcccgactgga | Testing of the plasmid construction pUCP24/T_fhaC for complementation |

**Table S2.** Growth curve parameters (K, Xint) and area under the curve (AUC).

|  | **Kp3380** | **Kp3380Δ*kpiD*** | **Kp3380Δ*fhaC*** | **Kp3380Δ*kpiD* Δ*fhaC*** | **Kp3380Δ*kpiD*_C** | **Kp3380Δ*fhaC*_C** |
| --- | --- | --- | --- | --- | --- | --- |
| K | 1.161 | 1.127 | 0.8437 | 1.072 | 1.070 | 0.6493 |
| Xint | 0.8612 | 0.8877 | 1.185 | 0.9326 | 0.9345 | 1.540 |
| AUC | 51.71 | 53.02 | 37.05 | 49.03 | 43.82 | 33.01 |
